# A Case of Gallbladder Adenocarcinoma Presenting as Mirizzi Syndrome in a Non-Jaundiced Patient With Recent Weight Loss

**DOI:** 10.7759/cureus.30459

**Published:** 2022-10-19

**Authors:** Lucia Soca Gallego, Kristina Fritz, Alvio J Dominguez, Maria F Castilla

**Affiliations:** 1 Dr. Kiran C. Patel College of Osteopathic Medicine, Nova Southeastern University, Fort Lauderdale, USA; 2 General Surgery, HCA Florida Fawcett Hospital, Port Charlotte, USA

**Keywords:** cholecystectomy, gallbladder adenocarcinoma, cholecystitis, gallstones, mirizzi syndrome

## Abstract

There are many different types of gallbladder diseases, mainly resulting from inflammation. The long-term presence of an insult to the gallbladder leads to chronic inflammation, which is a nidus for complications such as Mirizzi syndrome and gallbladder cancer, both of which can become mimics of one another. Preoperative diagnosis of either gallbladder cancer or Mirizzi syndrome is often difficult, leading to late diagnosis and complicating the patient's treatment course. We report a case of a 65-year-old male who presented with abdominal pain and significant weight loss, with no physical evidence of jaundice and normal liver function. This was initially diagnosed as acute cholecystitis and Mirizzi syndrome before being diagnosed as gallbladder adenocarcinoma on final histology.

## Introduction

Mirizzi syndrome is a rare complication of long-standing cholelithiasis with a <1% yearly incidence in developed countries. [1]. The pathophysiology leading to the different subtypes of Mirizzi syndrome includes a concrement impacted in the gallbladder’s cystic duct or infundibulum, leading to common hepatic duct obstruction, edema, and mechanical cholestasis. Clinical manifestations include abdominal pain, jaundice, and cholangitis [1,2]. Mirizzi syndrome leads to chronic inflammation and necrosis, which has been associated with gallbladder malignancy [1].

Gallbladder adenocarcinoma is an uncommon malignancy that is considered of low incidence in North America. The most common risk factor for gallbladder adenocarcinoma is chronic inflammation such as a history of cholelithiasis, gallbladder polyps, and primary sclerosing cholangitis [3]. Gallbladder adenocarcinoma and Mirizzi syndrome have similar clinical presentations that often make it difficult to diagnose preoperatively, thus increasing complication rates during surgical management [4].

We present a case of gallbladder adenocarcinoma presenting intra-operatively as Mirizzi syndrome in the setting of what appeared to be cholecystitis in a patient that did not have biliary obstruction or jaundice.

## Case presentation

A 65-year-old caucasian male with no significant past medical history presented to the emergency department complaining of worsening right upper quadrant (RUQ) abdominal pain associated with nausea and vomiting for the past three weeks. He endorsed a loss of appetite and weight loss of 50 lbs over the past three to four months. He also endorsed a recent history of easy bruising, nose bleeds, and hemoptysis of one week. The patient denied hematochezia, melena, and hematemesis. He was a former smoker and denied alcohol use. Physical examination was significant for scattered ecchymosis in bilateral lower extremities, diffuse abdominal pain, and negative for jaundice.

Labs on admission were notable for an abnormal WBC count of 14.3 k/uL with left shift, hemoglobin of 11 g/dL, and platelets of 35 k/uL. Liver function tests were also abnormal for alkaline phosphatase of 406 U/L and AST of 81 U/L and normal for ALT of 60 U/L. Serum bilirubin was normal at 0.31 mg/dL. Coagulation studies were abnormal for international normalized ratio (INR) of 1.9, prothrombin time (PT) of 21.8, and fibrinogen of 124.

Abdominal ultrasonography (US) revealed severe gallbladder wall thickening in the presence of gallstones. Common hepatic duct measuring 9 mm (normal <4 mm) and a mildly dilated common bile duct. Findings were strongly suggestive of acute cholecystitis.

**Figure 1:**
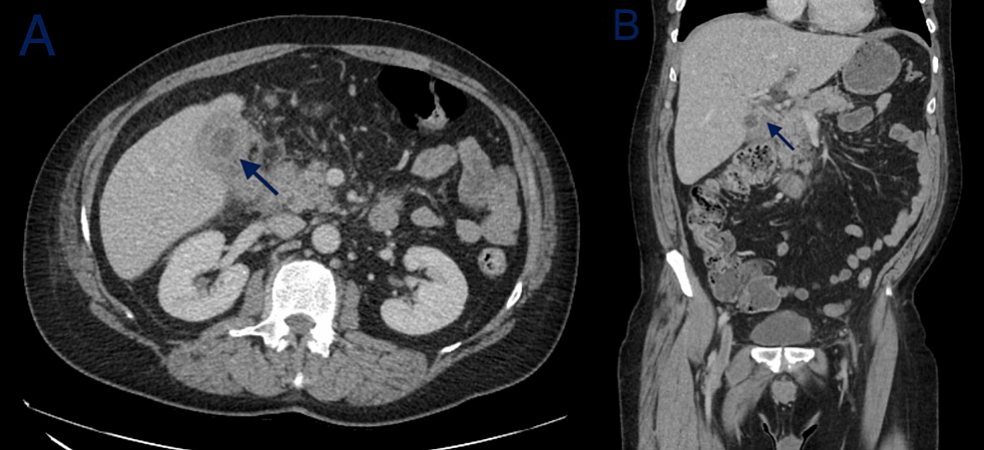
Contrast-enhanced CT images of the abdomen and pelvis (A) Axial and (B) coronal images showing extensive inflammation and diffuse moderate gallbladder wall thickening, with an extraluminal collection of fluid. There is effacement of pericholecystic fat with no calcified gallstones. Proximal common bile duct enhancement adjacent to inflamed gallbladder. Mildly enlarged lymph nodes in the gastrohepatic ligament.

Subsequent computed tomography (CT) of the abdomen and pelvis with intravenous contrast revealed gallbladder wall thickening, with an extraluminal collection of fluid versus a Phrygian cap with no calcified gallstones and proximal common bile duct enhancement adjacent to the inflamed gallbladder. In addition, findings were notable for the effacement of the pericholecystic fat and nonspecific mildly enlarged gastrohepatic ligament lymph nodes. See Figure 1.

Follow-up magnetic resonance cholangiopancreatography (MRCP) confirmed the presence of a large gallstone inside the gallbladder with narrowing of the proximal common duct secondary to mass effect from the adjacent inflamed gallbladder. There was also common bile duct wall thickening suggestive of cholangitis. The distal common bile duct and intrahepatic bile ducts were normal in caliber. An initial diagnosis of acute cholecystitis was made, with plans for cholecystectomy recommended.

Initially, the patient underwent robotic cholecystectomy, which was converted to open cholecystectomy in the setting of intractable bleeding. Notable intraoperative findings included a gallbladder that appeared stuck to the duodenum in the presence of a rock-hard cystic duct. In addition, the gallbladder appeared rock-hard with extensive dense adhesions extending to the transverse colon, duodenum, and omentum. Findings were also notable for a large stone removed from the area near the cystic duct. Immediately following the gallbladder removal, the wound was closed with drains in place, and the patient was admitted to the intensive care unit (ICU) in stable condition.

Following open cholecystectomy, the patient underwent re-exploration of the recent open abdomen due to worsening consumptive coagulopathy in the presence of bright-red blood per drain output that did not resolve with the correction of coagulopathy. Upon re-exploration of the abdomen, a moderate size hematoma of the colonic mesentery superior to the transverse colon with active oozing was identified, ligated, and irrigated. No evidence of bleeding was noted in the gallbladder fossa, and the abdominal cavity was closed.

The pathology report was remarkable for a grade 3 (G3) poorly differentiated primary gallbladder adenocarcinoma with lymphovascular and perimuscular connective tissue invasion without serosal involvement.

The patient was extubated on postoperative day six without further complications and had an otherwise uneventful recovery.

## Discussion

This is a case of Mirizzi syndrome complicated by gallbladder adenocarcinoma in an elderly patient without signs of obstructive pathology who presented with non-specific weight loss, coagulopathy, and new-onset abdominal pain. On presentation, cholecystitis was suspected, followed by an intraoperative diagnosis of Mirizzi syndrome and gallbladder adenocarcinoma on final histology.

There is a high coincidence of gallbladder adenocarcinoma in patients with Mirizzi syndrome. Risk factors associated with the development of Mirizzi syndrome include anatomical variations such as proximity of the common hepatic and cystic ducts, long and short cystic ducts, and chronic cholelithiasis [5]. In addition, Mirizzi syndrome also leads to chronic inflammation and necrosis. Chronic inflammation (i.e., from chronic cholelithiasis, certain infections, and biliary autoimmune conditions) has been associated with gallbladder carcinoma, as inflammation is a critical component that facilitates tumor progression [1,6]. Other risk factors associated with gallbladder adenocarcinomas include advanced age and female sex. Typically, the risk of developing this malignancy is highest with advanced age in women and people in Latin America, Asia, and Native Americans in the U.S.A. However, it is essential to maintain that anyone can develop gallbladder adenocarcinoma regardless of pre-existing risk factors. This patient's gallbladder adenocarcinoma likely developed secondary to chronic inflammation stemming from undiagnosed gallstone disease.

Mirizzi syndrome and gallbladder malignancy present similarly, with a broad clinical presentation ranging from asymptomatic to nonspecific symptoms of significant weight loss, generalized fatigue, obstructive jaundice, and abdominal pain. As a result, initial complaints of acute upper abdominal pain and/or jaundice are frequently evaluated with various diagnostic modalities such as RUQ US, endoscopic US (EUS), CT, MRCP, and/or endoscopic retrograde cholangiopancreatography (ERCP). In patients with possible Mirizzi syndrome, a US can reveal nonspecific findings of abrupt dilatation of the common bile duct above the gallbladder neck with possible gallstones. Diagnosis of Mirizzi syndrome is often confirmed with MRCP and/or ERCP, which can evaluate the extent of pericholecystic inflammation and extra biliary involvement, with the latter offering the ability to place a biliary stent [4]. In patients with possible gallbladder malignancy, the US can reveal nonspecific findings of wall thickening, calcification, and an intraluminal mass. EUS is a superior modality that evaluates for depth of tumor invasion and lymph node involvement. ERCP is helpful for preoperative planning, as it gives insight into tumor invasion of the intrahepatic duct or common bile duct [7]. Ultimately, despite advances in cross-sectional imaging, Mirizzi syndrome and early-stage gallbladder tumors are often not routinely encountered radiologically, resulting in significant diagnostic difficulties [3].

Currently, there is no standard of treatment for Mirizzi syndrome, given the varied clinical presentations. However, various classification systems attempt to describe the progression of Mirizzi syndrome to guide treatment. For instance, the new Csendes classification categorizes this case as type III Mirizzi syndrome due to the presence of a cholecystobiliary fistula with extensive bile duct wall erosion. As a result, promoting careful dissection of the gallbladder and the adjacent viscera to prevent complications [3]. Current guidelines also recommend evaluation and intervention with ERCP and stent placement when appropriate, proceeded by open cholecystectomy for better visualization of the anatomy [4].

Current guidelines for managing gallbladder malignancy diagnosed preoperatively include cholecystectomy with local resection of regional lymph nodes. In the event of incidental postoperative diagnosis on tissue histology with T2 or higher staging, guidelines recommend possible re-exploration of the abdomen. Adjuvant therapy is recommended in the setting of malignancy with positive lymph nodes or a T2 stage or higher with six months of chemotherapy or four months of chemoradiation in addition to frequent follow-up with oncology specialists [6]. Ultimately, the management approach needs to be reviewed in accordance with individual patient presentation and disease progression.

## Conclusions

Chronic gallbladder inflammation, most commonly resulting from gallstones, as was the case in this patient, can lead to complications such as Mirizzi syndrome and gallbladder adenocarcinoma. Late diagnosis often complicates the treatment course of patients. Maintaining a high level of suspicion for malignancy in the setting of cholecystitis presenting with symptoms of significant weight loss, easy bruising, and coagulopathy in non-jaundiced, previously asymptomatic patients can help clinicians plan pre-operatively and individualize treatment to minimize complications and improve patient outcomes and quality of life.
